# Surgical Anatomy

**Published:** 1852-01

**Authors:** 


ARTICEL II.

*Surgical Anatomy.* By JOSEPH MECLAISE, Surgeon; with colored plates, parts 4 and 5. Philadelphia: Blanchard & Lea, 1851. (From the Publishers through S. C. Griggs & Co., Chicago.)

These parts close the work, which has been issued in numbers by the American Publishers. The two parts treat of the illiac, femoral, and perinæal regions and of the inferior extremities.

As a work of utility and art of its kind we can, without hesitation, speak of it in terms of the highest praise. The surgeon will find it one of the most useful and elegant works that he can obtain for his library, and can purchase it for what we should think a very moderate price, being but nine dollars for the work complete.

